# Occupational health risk perceptions and determinants: knowledge and attitude of sanitary workers in public hospitals of Ethiopia

**DOI:** 10.3389/fpubh.2025.1509595

**Published:** 2025-05-09

**Authors:** Sina Temesgen Tolera, Tesfaye Gobena, Abraham Geremew, Elka Toseva, Nega Assefa

**Affiliations:** ^1^College of Health and Medical Sciences, Haramaya University, Harar, Ethiopia; ^2^Department of Hygiene, Faculty of Public Health, Medical University of Plovdiv, Plovdiv, Bulgaria

**Keywords:** attitude, determinants, occupational health, knowledge, risk, sanitary workers

## Abstract

**Introduction:**

Occupational health risks (OHRs) perception refer to an individual’s subjective assessment of the likelihood and severity of potential health hazards within their workplace, which are influenced by their knowledge and attitude. The OHRs perceptions of sanitary workers (SWs) in Ethiopia have not yet been well studied. Therefore, this study aims to assess the knowledge and attitude of SWs about OHR perceptions and their determinants in public hospitals of eastern Ethiopia.

**Methods:**

A hospital-based cross-sectional study was conducted on a total of 809 SWs, which comprised face-to-face interviews. Knowledge items were measured by [YES/NO] and then categorized as “good” if they scored 16–20 points; “fair” if 10–15 points; and “poor” if < 10 points. On the other hand, attitude items were measured on Likert scales [strongly disagree (1) to strongly agree (5)] and classified as level 1: unfavorable; level 2: neutral; and level 3: favorable. Stata 17MP version was used for data analysis. The univariate analysis was applied for frequency, prevalence, media, and mean. Multilevel ordinal logistic regression was conducted for the predictions. Structural equation modeling (SEM) was used to determine the correlations between level of knowledge of and attitude toward OHRs perceptions as well as with their predictors.

**Result:**

This study found that the poor level of knowledge of and unfavorable attitude toward OHRs among SWs were 67.35 and 42.66%, respectively. The difference in knowledge of and attitude toward OHRs perceptions of SWs between hospitals was 19.34 and 39.55%, respectively. The final model showed that the variables trained on occupational health and safety [OHS] (AOR: 4.90; 3.10, 7.75), satisfied with job (AOR: 1.88; 1.10, 3.75), and satisfied with environment (AOR: 2.57; 1.09, 6.05) were significantly associated with higher knowledge levels about OHRs. However, SWs who were satisfied with environment (AOR: 2.67; 1.03, 6.92) and who follow good infection prevention and control (IPC) practice (AOR: 20.43; 15, 35.84) were significantly associated with a high level of attitude toward OHRs. SEM results showed that OHS training (*β*: 0.35; 0.27, 0.44) and compliance with IPC (*β*: 0.07; 0.02, 0.12), as well as compliance with personal protective equipment (*β*: 0.14; 0.04, 0.23), had a positive impact on the knowledge and attitude about OHRs.

**Conclusion:**

This study concluded that the majority of SWs had inadequate knowledge about and negative attitude toward OHRs perceptions. The following variables played the most significant role in predicting the SWs’ knowledge of and attitude toward OHRs: OHS training, job and environmental satisfaction, safety measures, and job stress, which could be considered for further interventions.

## Introduction

Occupational health risks (OHRs) perception in terms of knowledge and attitude refers to an individual’s subjective assessment of the likelihood and severity of potential health hazards within their workplace (1). An individual’s OHRs perceptions could be influenced by various factors such as their knowledge, attitude, experience, personal beliefs, and the perceived control they have over potential risks, ultimately determining how they behave in relation to safety precautions (2). OHRs perceptions can be broadly categorized into halo effect perceptions (HEPs) and fundamental attribution error perceptions (FAEPs). This study found that negative information regarding potential health hazards within healthcare facilities can lead to a negative halo effect (3). FAEPs, where individuals attribute others’ behaviors toward internal factors (such as personality) rather than situational factors, can lead to misunderstandings (4).

OHRs perceptions are also expressed as self-serving bias perceptions (SBPs), projection perceptions (PPs), stereotyping perceptions (SPs), and selective perceptions (SP). SBPs posit that people tend to have positively biased perceptions toward themselves by ascribing failures to external factors to overcome dissatisfaction with their capability to complete a certain task (5). PPs are used when the world is often a reflection of our own inner thoughts, feelings, and experiences, rather than objective realities (6) On the other hand, SPs are used for forming generalized, often inaccurate beliefs about things (7) Furthermore, selected perception (SP), which is used in this study, shows a tendency to selectively interpret what individuals see based on background, experience, knowledge, and attitude (8). In general, OHR perceptions have two dimensions: cognitive dimension, which relates to how much people know about and understand risks, and emotional dimension, which relates to how they feel about them (9).

Literature on job environments suggests that good knowledge about OHRs, normal behavior, favorable attitude toward OHRs, and compliance of occupational health and safety (OHS) can reduce the risk of accidents and injuries in the workplace (10). This is because good knowledge refers to the state where employees are well informed about potential hazards, safety protocols, and proper use of equipment, and they are better equipped to identify and avoid risks in their work environment (11). A positive attitude toward safety encourages employees to actively participate in safe practices, report hazards, and pay attention to the safety of themselves and their colleagues. Furthermore, when safe practices become ingrained in routine work habits, i.e., when they become normal behavior, the likelihood of risky behaviors that could lead to accidents is reduced (11, 12).

However, in unsafe and unhygienic work conditions, sanitary workers (SWs) are exposed to various risk factors due to the lack of OHR knowledge and attitude (13). Risk perception of SWs refers to their subjective judgments about the likelihood of OHR through the cognitive dimension of OHRs, which relates to how much people know about and understand risks, and its emotional dimension, which relates to how they feel about them (9). In addition, low risk perception refers to the cognitive bias in which individuals underestimate potential risks present in their work environment (14). In brief, risk perception refers to an individual’s spontaneous risk assessment, reflecting public attitude toward or beliefs about a potential harm (15, 16). In general, higher levels of perceived risk are related to a lower tendency to engage in risky behavior (17).

Previous studies have reported that poor knowledge about and unfavorable attitude toward health and safety are the factors with the most negative effects on OHRs perceptions of hospital staffs, particularly among SWs (18, 19). On the other hand, a lack of understanding about and unfavorable attitude toward OHR are major obstacles to compliance with health and safety measures (20). According to the “knowledge–attitude–practice model,” changes in human behavior occur in three stages: knowledge acquisition, belief generation, and behavior formation (21). Acquiring the appropriate information and good knowledge about and attitude toward the workplace makes it easier for employees to take necessary steps regarding their welfare (22).

A study carried out in Dhaka, Bangladesh, has shown that the majority of SWs have low OHRs perceptions regarding the chances of being injured at work. More than one-third of the participants believed that infection, injuries, accident, and death can happen to anyone, at any time, and anywhere (23). Another study conducted in Kenya has revealed that the majority of the participants [70%] had little knowledge about OHRs and its problems (24). Similarly, another study has also reported individuals’ lack of awareness of risk inherent in their jobs (25). In addition, a study conducted in Ethiopia has found that 39.2% of SWs did not know about work-related risks and approximately 36.9% of them did not know how to prevent risks (26).

A previous study has found that the knowledge of SWs on nosocomial infection and OHRs was extremely poor (27). Furthermore, another study has found that 23.6% of SWs had an unfavorable attitude toward risks and 91.9% of them had a poor practice of risk preventions (26). Besides, a lack of understanding of and unfavorable attitude toward OHRs have been found to be increased among all workers, especially among SWs, which has led to a low perception of risk. For instance, almost 77% of SWs were unaware of the possibility of occupational infections in hospitals. This study further revealed that approximately 20% of SWs were unaware of the health concerns associated with their workplace and had an unfavorable attitude toward the risk of contracting hepatitis via blood waste in hospitals (27). Some studies have reported that SWs have a low perception of risk susceptibility (28), low awareness about occupational risk (29), and low perception about acquiring infections (30).

As reported in numerous studies, a few factors have been associated with unfavorable attitude toward and poor knowledge about OHRs among SWs. For example, male gender and high work experience (31), and being educated and trained on OHS (32) were more likely associated with low OHRs. The factors environment dissatisfaction, job dissatisfaction, and job stress were more likely associated with high OHRs (33). In addition, behavioral factors such as alcohol consumption, sleeping disorder, *khat* chewing (local name of a substance with a green leaf in use in Ethiopia) and cigarette smoking (34, 35), and less attention to OHS service at the institution level (36) could lead to low knowledge of and unfavorable attitude toward OHRs. Furthermore, lack of supervision, weak infection prevention and control (IPC) practice, work overload (37), poor social recognition (38), and mental health problems (39) were significantly associated with low knowledge of and unfavorable attitude toward OHRs. However, to date, no study has been conducted on the level of knowledge of and attitude toward OHRs and their determinants among SWs in hospitals in Ethiopia. Therefore, this study aims to assess OHR perceptions in terms of knowledge, attitude, and determinants among SWs in public hospitals of eastern Ethiopia.

## Methods

### Study design and settings

**Figure 1:**
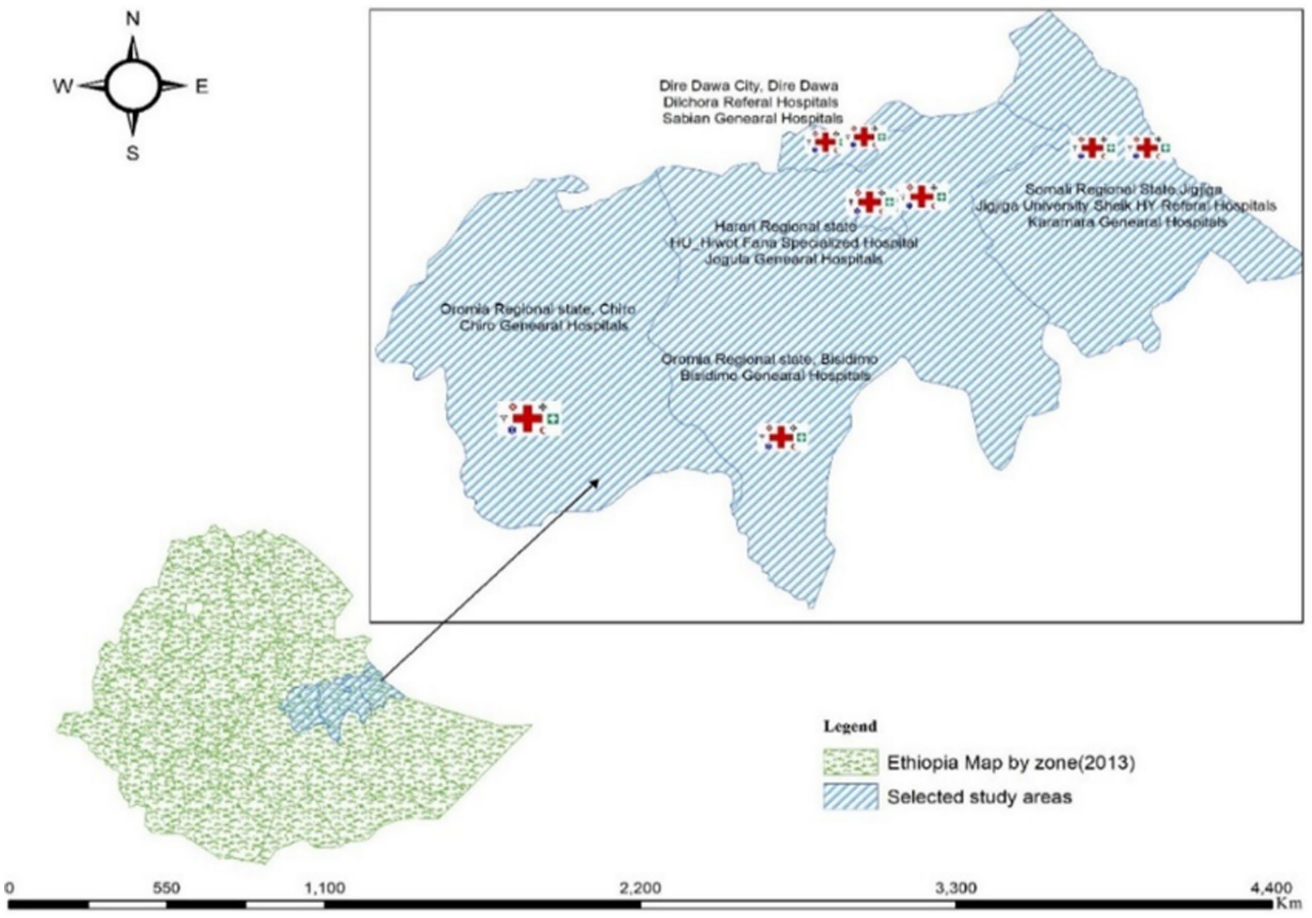
Selected study areas of eastern Ethiopia.

This hospital-based cross-sectional study was conducted in public hospitals in eastern Ethiopia from April to August 2023. The study region included one city administration and three regional states in eastern Ethiopia. Among 14 hospitals, eight were selected via random sampling, two each from the four studied regions (Figure 1).

### Study population

All SWs working in hospitals across eastern Ethiopia were the source of the study population. All SWs working in wards, those involved in collecting and emptying latrine/toilet waste, either permanent or outsourced, and those with more than 1 month of work experience were included. However, only SWs employed in public hospitals in eastern Ethiopia were the targeted units of the study. Moreover, SWs with less than 1 month of experience and those on annual and maternal leave during the study period were excluded.

### Sample determination

The prevalence of the level of knowledge about and attitude toward OHRs among SWs in public hospitals of Ethiopia was calculated using the following single-proportion formula:


$$N=\frac{z^{2}pq}{d^{2}}$$

,where

- N is the required sample size,
- *Z* is the reliability coefficient at 95% confidence interval (1.96),
- *p* is the population proportion,
- q is equal to 1 − *p*, and
- *d* is the acceptable error (0.05).

The previous prevalence of low knowledge about the risks [70%] found from Kenya (24) and prevalence of unfavorable attitude to the risks, found Ethiopia (27) were computed for this formula. Hence, the sample size for knowledge of OHR was calculated as follows: $\left[ni\right]=\frac{{\left(1.96\right)}^{2}\;\left(0.3\right)\left(0.7\right)}{{\left(0.05\right)}^{2}}=323$ . The sample size for attitude toward was calculated as follows: $\left[ni\right]=\frac{{\left(1.96\right)}^{2}\;\left(0.236\right)\left(0.764\right)}{{\left(0.05\right)}^{2}}=277$ . The sample size of knowledge variables (323) was higher than that of attitude variables (277). Hence, the sample size had to be increased to obtain sufficient and precise information. Therefore, a design effect of 2.0 was used to increase the sample size. Thus, the sample size became 646, which was approaching the total number of SWs actively working (*n* = 809) in the eight hospitals. Thus, finally, all of them were recruited for this study.

### Sampling procedures

**Figure 2:**
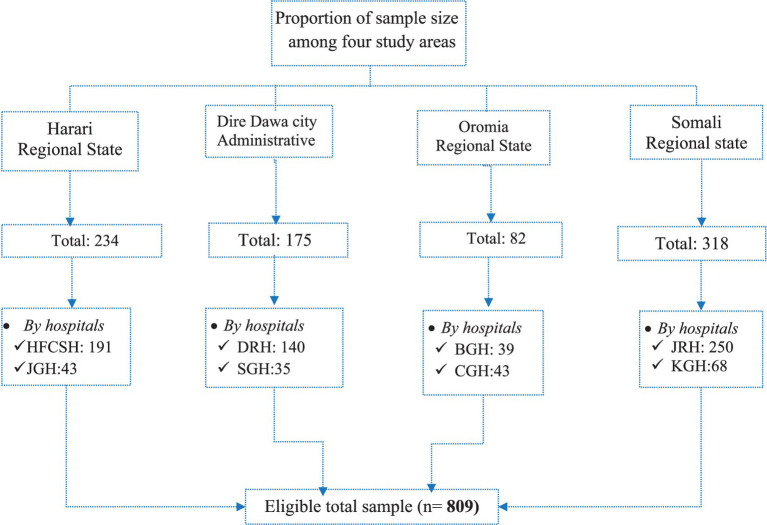
Schematic representation of the sampling procedure of SWs in the selected public hospitals. HUCSH, Haramaya University Comprehensive Specialized Hospital; JGH, Jugola General Hospital; DRH, Dilchora Referral Hospital; SGH, Sabain General Hospital; JUSHRH, Jigjiga University Referral Hospital, KGH, Karamara General Hospital; BGH, Bisidimo General Hospital; CGH, Chiro General Hospital.

General and referral hospitals in four regional states were included in this study. A total of 234, 175, 82, and 318 SWs were recruited obtained from hospitals in the Harari regional state, Dire Dawa city, Oromia regional state, and Somali regional state, respectively, and questionnaires were distributed to each hospital based on the number of SWs participating in the study (Figure 2).

### Study variables

Among study variables, the dependent variables consisted of knowledge of and attitude toward OHRs, adapted from a previous study (40). Independent variables included common socio-demographic variables such as age, sex, educational status, work experience, job categories, marital status, and monthly income. In addition, institutional variables such as providing OHS training and following IPC practice were included. The safety variable utilization of personal protective equipment (PPE) was also included. Furthermore, behavioral variables such as alcohol consumption, cigarette smoking, chewing *khat*, and sleeping disorder, and working environment variables such as environment satisfaction, working hours, job satisfaction, job stress, workload, and social recognition were included. The procedures followed for data analysis were discussed in Data Collection Tools.

#### Data collection tools and procedures

##### Data collection tools

A structured, standard, and closed-ended questionnaire was prepared. The assessment tool is presented in Supplementary material 1, with the consent of participants. Epistemological philosophy (41) was used to assess SWs’ knowledge and understanding of OHRs, as well as their attitude toward OHR, in accordance with a previous study (11). The aim was to explore their perceptions, feelings, thoughts, beliefs, expectations, and behavior toward OHRs, which was extremely significant for preventing or mitigating risk, following a previous study (42). Taking this into account, questions were constructed addressing knowledge items, attitude items, and other related aspects.

##### Knowledge items

Ten standard questions based on the Boolean logic (YES (1)/NO [0]) were prepared to elicit what SWs know about or aware of and assess their knowledge about and attitude toward OHRs, in accordance with a previous study (41). Based on the scores obtained, knowledge was classified into poor (<10 scores or < 50%), fair (10–15 scores or 51–75%), and good knowledge (>15 scores or >75%), following a previous study (43).

##### Attitude items

Ten standard questions were developed to assess SWs’ perceptions, feelings, thoughts, beliefs, expectations, and behavior toward OHR, in accordance with previous studies (44, 45). The items were categorized as perceived susceptibility, benefits, severity, and safe, following the protocol of a previous study (46). Attitude items were evaluated using Likert scales 1–5 [5, strongly agree to 1, strongly disagree], which is adapted from a previous study (47). Then, the five-point Likert scale was reduced to a three-point one (level I–III), based on a previous study (48). Among the 10 items, level I (unfavorable attitude) included those scored 1.00–2.99 out of 10 items; level II (neutral attitude) 3.00; and level III (favorable attitude) 3.01–5.00.

##### Questions on associated factors

For this purpose, 18 questions were prepared, which were categorized as follows: behavioral factors: to assess sleep disorders, heavy alcohol consumption, chewing *khat*, and smoking cigarette, standard questions were prepared using Boolean logic YES (1) and NO [0], which was adapted from a previous study (49); and institutional factors: to assess institutional factors such as supervision, OHS training, adequate PPE supply, and work shift, standard questions were prepared using Boolean logic YES (1) and NO [0], in accordance with a previous study (50).

*Job and environment satisfaction: Job satisfaction* is a subjective response of study participants about their job to the question of whether it is pleasurable or not, whereas *environment satisfaction* primarily mainly refers to the safety, comfort, and harmony of the objective hospital environment in which they work, excluding salary and promotion factors, based on a previous study (51). *Job stress symptoms* were evaluated using subjective responses of respondents to the question of whether they feel stressed due to the job or not, in which higher values indicated higher psychological stress (52). *Workload, IPC practice, and work shift:* A single item for each factor was prepared, and the SWs were asked to answer either YES (1) or NO [0], following a previous study (53).

#### Collection pattern

All hospital SWs worked at any of the following three shifts in a week: the first shift starts at 7:00 a.m. and ends at 12 a.m. (morning); the second shift starts at 1:00 p.m. and ends at 5:00 p.m. (afternoon); and the third shift (night) starts at 12 p.m. to 6:00 p.m. (SWs were assigned this shift for not more than two consecutive days). By considering the shift timings, the questionnaires were administered between 9:00 and 10:00 a.m. for shift 1 and between 3:00 and 4:00 p.m. for shift 2. The same procedure was followed for shift 3 after 2 days.

#### Data collectors

Four individuals with a master of environmental health degree, two with a master of occupational health and safety, and two with a master of public health were recruited for data collection. Four supervisors were assigned to the eight hospitals during the data collection period.

### Data quality

Literature on adherence to OHS requirements of SWs served as the basis for the designed questions. Therefore, to guarantee the quality of the data, the first task was to create standard, structured surveys in English that included closed-ended questions. Then, they were translated into three local languages. The second task was assigning professional data collectors. The third task was providing appropriate training to data collectors and supervisors. The fourth task was evaluating the reliability and validity of the items (prepared questions) for internal consistency. Reliability analysis was carried out to ensure consistent measurement across time and across various items, in accordance with a previous study (54). Validity analysis was conducted by evaluating appropriate words and concepts using a statistical model (55). The fifth task was conducting a pretest study (5%) outside of study areas, “at *Haramaya General Hospital*,” prior to the main study, which was aimed at avoiding the uncertainties of the data collection instruments, as well as ensuring the feasibility, clarity, and precision of the questionnaire.

### Data analysis

Data were coded and entered into Epi Data version 3.1 (The EpiData Association” Odense, Denmark). Stata 17 Mp version (StataCorp LP in College Station, Texas). Then, they were exported to then, data was exported to Stata 17MP version for analysis. Descriptive statistics were used to characterize independent and dependent variables. Meanwhile, a multilevel ordinal logistic regression model was used for predictors and determinants of categorical variables. Four models were performed: model 0 (null model), model 1 (within-group individuals, SW variables), model 2 (between-group individuals, hospital variables), and model 3 (a combination of models 1 and 2). However, since this study has a number of tables, tables for models 1 and 2 are not included. The value of the intraclass correlation coefficient (ICC) was set according to previous studies. If the ICC is greater than 0.05, it is generally recommended to use a threshold of a multilevel model to account for the clustering effect within groups depending on the research field and specific context. In the present study, the value of the ICC for the outcomes at null hypothesis (only outcome) higher than 10% was used according to Wilms and Lanwehr (56). A mixed-effect model was used to estimate the regression coefficient (observable parameters). Akaike’s information criterion (AIC) and Bayesian information criterion (BIC) were used for model comparison. A higher difference in either AIC or BIC indicates stronger evidence for one model over the other (the lower the better), which was presented in each multilevel analysis table. Likelihood ratio (LR) of Chi_2_ -*p*-value also computed for the model test, which was less than *p*-value of 0.05. Then, the model with the highest LR was selected. Sensitivity and specificity of the model were also tested for each dependent variable to evaluate the model’s ability to predict true positives and true negatives, respectively. The Crude odds ratio (COR) and adjusted odds ratio (AOR) of variables along with a 95% confidence interval (CI) were presented by tables. Independent variables with a *p*-value of 0.20 were selected for the final multivariable analysis. Variables with AOR and 95% CI at a *p*-value<0.05 were reported. The COR and AOR with a 95%CI were presented at model 3. Multicollinearity was also examined using the variance inflation factor (VIF), which measured how an independent variable’s variance was inflated, with a cutoff point of less than 10. Hosmer–Lemeshow (HL) goodness-of-fit test was also used for model fit, where variables with a small chi-square value and a high *p*-value closer to 1 were accepted, based on a previous study (57). Moreover, variables with an AOR at a *p*-value of <0.05 in the multivariable multilevel ordinal logistic regression analysis were selected for structural equation modeling (SEM), which was used to evaluate the correlation of knowledge and attitude toward OHRs as well as with their corresponding predictors.

## Results

### Socio-demographic characteristics

Out of the 809 SWs, 729 (90.11%) were eligible for this study. The mean ± SD values for age, job experience, educational status, and monthly income salary were 34.35 ± 7.60, 6.65 ± 6.36, 6.78 ± 2.51, and 36.32 ± 6.68 USD, respectively (Table 1).

**Table 1:** Socio-demographic status of SWs in selected public hospitals in eastern Ethiopia.

Socio-demographic variable	Classification	Frequency (no.)	Percentage	Mean ± SD
Sex	Female	718	98.49	
	Male	11	1.51	
Age (years)	≤ 24	63	8.64	
	25–35	350	48.01	34.35 ± 7.60
	> 35	316	43.35	
Work experience (years)	≤2	133	18.24	
	3–5	288	39.51	6.65 ± 6.36
	> 5	308	42.25	
Educational status (grade)	≤ Grade 4	160	22.07	
	Grade 5–8	283	39.03	6.78 ± 2.51
	>Grade 8	282	38.90	
Marital status	Single	142	19.48	
	Married	506	69.41	
	Separated	59	8.09	
	Divorced	22	3.02	
Monthly income (USD)	≤ $20.15USD*	12	1.65	
	$20.16–42.95**	672	92.18	36.32 ± 6.68
	> $42.95USD	45	6.17	
Job categories	Cleaners	679	93.14	
	Waste collectors	50	6.86	
Employment type	Permanent	709	97.26	
	Contracts	20	2.74	
Job rotation	Shift 1	360	49.38	
	Shift 2	262	35.94	
	Shift 3	106	14.54	

Level salary I*($20.15 = 1100ETB); Level V** ($42.95 = 2344ETB), Levels of civil service salary, Ethiopia Job Evaluation and Grading [JEG], 2019 [Where 1 dollar ($) = 54.58 ETB, September 2023].

### Knowledge and determinants of occupational health risks

The percentage of good, fair, and poor levels of knowledge about OHRs among SWs was 12.21% (*n* = 89), 20.44% (*n* = 149), and 67.35% (*n* = 491), respectively. The multivariable multilevel ordinal logistic regression model showed that being female (AOR: 0.39; 0.10, 0.87), working in shift 2 (AOR: 0.67; 0.43, 0.96) unfavorable attitude toward OHRs (AOR: 0.33; 0.17, 0.66), and poor IPC practice (AOR: 0.35; 0.20, 0.62) decreased the level of knowledge about OHRs. Those who received OHS training (AOR: 4.90; 3.10, 7.75), who were satisfied with their job (AOR: 1.88; 1.11, 3.75), and who were satisfied with environment (AOR: 2.57; 1.09, 6.05) showed an increased level of knowledge about OHRs (Table 2).

**Table 2:** Multilevel ordinal logistic regression model for predictors of knowledge level about occupational health risks among SWs from selected public hospitals.

Variables with categories		Knowledge about risk (N:729)			COR (95% CI)	AOR (95% CI)
		Good: 89	Fair: 149	Poor: 491		
Sex	Male	2(18.18)	4(36.36)	5(45.45)	1	1
	Female	87(5.01)	145(20.19)	485(74.79)	0.17[0.05–0.53] ^**^	0.39[0,1,0.97] ^*^
Attitude toward risk	Favorable	43(13.15)	92(28.13)	192(58.72)	1	1
	Neutral	28(6.85)	53(13.00)	325(79.46)	0.38[0.25, 0.58] ^**^	0.50[0.29,0.89] ^*^
	Unfavorable	18(39.13)	4(8.70)	24(52.17)	0.23[0.14, 0.40] ^**^	0.33[0.17,0.66] ^*^
Job stress	Yes	40(18.96)	72(34.12)	99(46.92)	1	1
	No	49(9.46)	77(14.86)	392(75.8)	0.25[0.15,0.41] ^**^	0.35[0.19,0.65] ^*^
IPC practice	Good	28(13.15)	95(44.60)	90(42.25)	1	1
	Fair	21(8.05)	50(19.16)	190(72.80)	0.23[0.16,0.35] ^**^	0.35[0.20,0.62] ^*^
	Poor	40(15.67)	4(1.57)	211(82.75)	0.13[0.08,0.20] ^**^	0.39[0.20, 0.76] ^*^
Job satisfaction	No	49(7.62)	123(19.13)	471(73.25)	1	1
	Yes	40(46.51)	26(30.23)	20(23.26)	0.36[0.26,0.51] ^**^	1.88[1.11,3.75] ^*^
Trained on OHS	No	36(8.33)	33(7.64)	363(84.03)	1	1
	Yes	53(17.85)	116(39.06)	128(43.10)	7.9[5.43, 11.68] ^**^	4.90[3.10,7.75] ^*^
Environmental satisfaction	No	49 49(7.33)	137(20.51)	482(72,16)	1	1
	Yes	40(65.57)	12(19.67)	9(14.75)	3.41[2.16,5.36] ^**^	2.57[1.09, 6.05] ^*^
Hazard exposure	No	26(9.811)	48(18.11)	191(72.08)	1	1
	Yes	53(11.67)	101(22.24)	300(66.08)	0.50[0.35,0.72] ^**^	0.43[0.27,0.68] ^*^

Model summary	ICC	AIC	BIC	Likelihood ratio	Sensitivity	Specificity
Level 1	19.64%*	853.90	913.53	0.09	38.17%	92.39%
Level 2	18.88%*	797.51	861.71	0.15	55.32%	91.68%
Level 3	19.34%*	780.38	890.45	0.19	60.22%	91.28%

At model 3: *Statistically significant at 0.05 and ** ≤ 0.20; ICC: 19.34%. The variance of knowledge about occupational health risks from hospital to hospitals was 19.34%; Hosmer and Lemeshow goodness *X*^2^ (8) = 9.50, *p*-value = 0.30, VIF: 2.00, Pseudo *R*^2^ = 0.2343.

### Attitude toward occupational health risk

The percentage of unfavorable attitude toward OHRs among SWs was 42.66%. Perceived attitude of sustainability, perceived benefit of workplace, perceived severity of workplace, and perceived safe environment toward OHRs comprised 363 (49.79%), 134 (18.38%), 252 (34.57%), and 363 (49.79%), respectively (Table 3).

**Table 3:** Attitude of sanitary workers toward occupational health risks among public hospitals of eastern Ethiopia.

Type of attitude perceived	Alpha Cronbach	Median	Unfavorable Freq. (%)	Neutral Freq. (%)	Favorable Freq. (%)
Perceived sustainability of risk at the workplace (*n* = 3)	0.82	3.00	363(49.79)	121(16.60)	245(33.61)
Perceived benefit of risk at the workplace (*n* = 3)	0.75	3.00	134(18.38)	81(11.11)	514(70.51)
Perceived severity of risk at the workplace (*n* = 2)	0.70	3.00	252(34.57)	122(16.74)	355(48.70)
Perceived safe environment of risk at the workplace (=2)	0.70	3.00	363(49.79)	106(14.54)	260(35.67)
Overall	0.73	3.00	311(42.66)	28(3.84)	390(53.50)

### Attitude and determinants of occupational health risks

Multivariable multilevel ordinal logistic regression model revealed that SWs who had positive perceptions about susceptibility to risk (AOR: 10.44, 95%CI: 5.93, 18.39), who had positive perceptions about severity to risk (AOR: 48.14, 95%CI: 26.58, 21), who worked in shift 2 (AOR: 2.57, 95%CI: 1.35, 4.87), who did not experience workload (2.55, 95%CI: 1.04, 6.38), who were satisfied with the work environment (AOR: 2.67, 95%CI: 1.03, 6.92), who were susceptible to risk (AOR: 10.44, 95%CI: 5.93, 18.39), who followed good IPC practice (AOR: 20.43; 15.00, 35.84), and who received OHS training (AOR: 3.45; 95%CI: 1.51, 6.22) were more likely to show better attitude toward OHRs than others (Table 4).

**Table 4:** Multilevel ordinal logistic regression model for predictors of attitude toward occupational health risks among SWs from selected public hospitals.

Categories of variables		Attitude toward risks (*N* = 729)			COR (95% CI)	AOR (95% CI)
		Favorable: 39	Neutral: 28	Unfavorable:311		
Susceptible to risk	Negative	113(28.97)	10(35.71)	240(77.17)	1	1
	Neutral	76(19.49)	6(21.43)	39(12.54)	3.96[2.59, 6.05] ^**^	4.19[2.23, 7.88]
	Favorable	201(51.54)	12(42.86)	32(10.29)	10.91[7.72, 20.67] ^**^	10.44[5.93, 18.39] ^*^
Severity of risk	Negative	26(6.67)	11(39.29)	215(69.13)	1	1
	Neutral	50(12.82)	11(39.29)	61(19.61)	5.72[3.51, 9.34] ^**^	3.40[1.90, 6.10]
	Favorable	314(80.51)	6(21.43)	35(11.25)	60.41[37.79, 80.32] ^**^	48.1[26.58, 58.21] ^*^
Presence of workload	Yes	96(24.62)	3(10.71)	68(21.86)	1	1
	No	294(75.38)	25(89.29)	243(78.14)	1.67[0.32, 0.89] ^**^	2.55[1.04, 6.38] ^*^
PPE practice	Yes	199(51.03)	13(46.43)	129(41.48)	1	1
	No	182(58.52)	15(53.57)	159(46.63)	0.69[0.51, 0.92] ^**^	0.53[0.38, 0.84] ^*^
Social recognition	Yes	280(71.79)	15(53.57)	119(38.26)	1	1
	No	110(28.21)	13(46.43)	192(61.74)	0.28[0.21, 0.37] ^**^	0.53[0.37, 0.77] ^*^
Knowledge of risk	Poor	325(83.33)	24(85.71)	192(61.74)	1	1
	Fair	53(13.33)	4(14.29)	92(29.58)	0.34[0.23, 0.49] ^**^	0.62[0.37, 0.89]
	Good	12(3.08)	0(0.00)	27(8.68)	0.24[0.11, 0.52] ^**^	0.45[0.19, 0.95] ^*^
IPC practice	Poor	62(15.90)	5(17.86)	130(41.80)	1	1
	Fair	151(38.72)	14(50.00)	99(41.83)	0.32[0.22, 0.47] ^**^	0.29[0.08, 0.89]
	Good	177(45.38)	9(32.14)	82(26.37)	11.12[1.00, 2.00] ^**^	20.43[15.98, 35.84] ^*^
OHS training	No	276(70.79)	17(60.71)	139(44.69)	1	1
	Yes	114(29.23)	11(39.29)	172(55.31)	2.11[0.30,0.54] ^**^	3.45[1.51, 6.22] ^*^
Environment satisfaction	No	352(90.26)	25(89.29)	291(93.57)	1	1
	Yes	38(9.74)	3(10.71)	20(6.43)	1.52[0.89, 2.58] ^**^	2.67[1.03, 6.92] ^*^

Model summary	ICC	AIC	BIC	LR	Sensitivity	Specificity
Level 1	37.83%*	964.00	1005.33	0.03	68.11%	82.39%
Level 2	33.74%*	911.97	962.48	0.08	75.42%	81.68%
Level 3	39.55%*	910.24	983.70	0.09	80.72%	91.28%

At model 3: *Statistically significant at 0.05 and ** ≤ 0.20; ICC 39.55%, indicating the difference in attitude about occupational health risks among SWs from one hospitals to another; LR (Likelihood ratio): 0.093, indicating that the deviance of model 3 from model 0 was 0.093; Pseudo *R*^2^ = 0.1614; Hosmer and Lemeshow *X*^2^(8) = 18.94, *p*-value = 0.0552; VIF = 3.62.

### SEM of knowledge of and attitude toward risks

**Figure 3:**
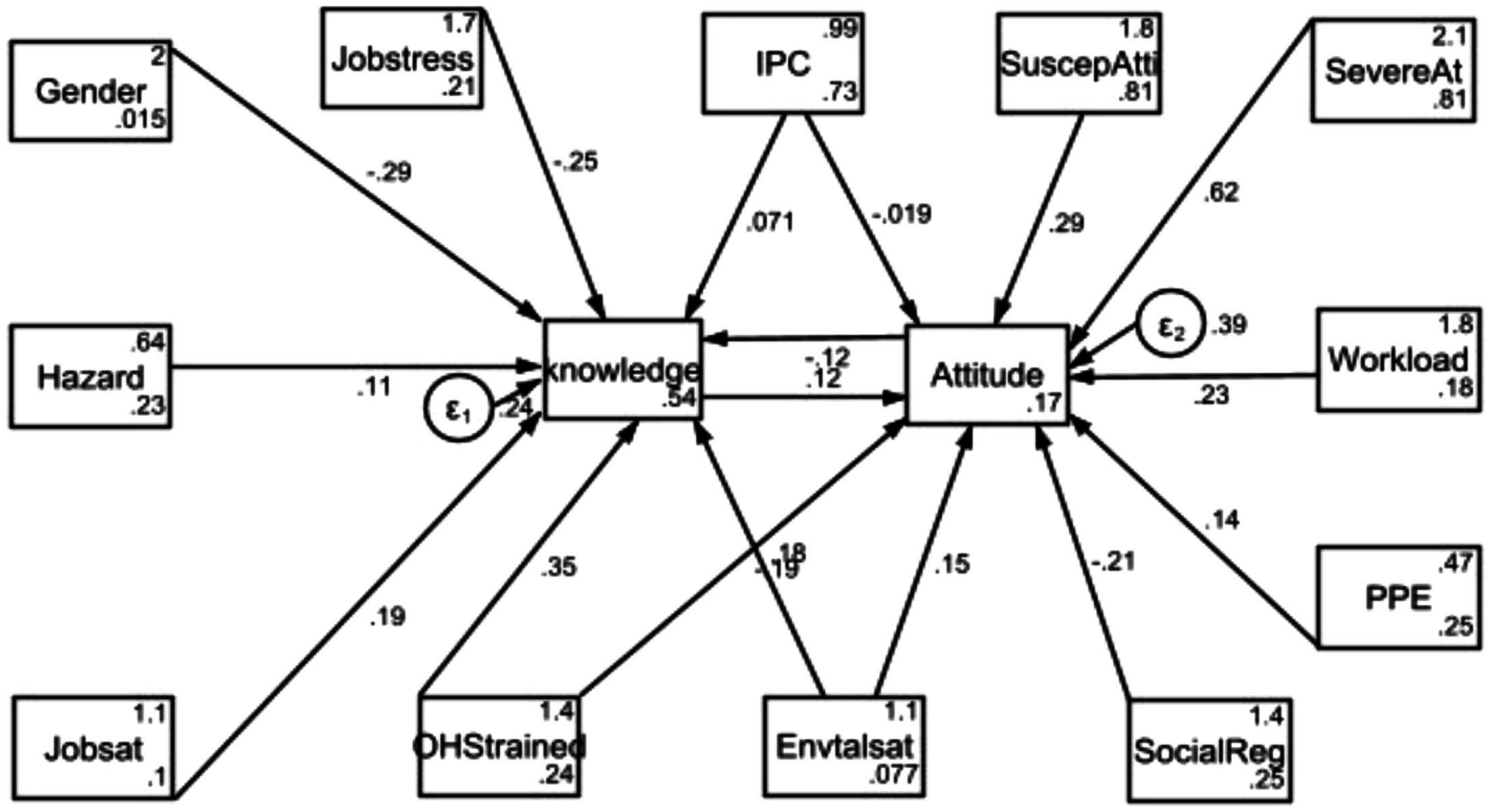
Schematic showing significant independent variables for AOR of knowledge and attitude toward occupational health risk perceptions among SWs in public hospitals. IPC: Infection prevention and control; PPE: Personal protective equipment; SuscepAt: Susceptibility of attitude to risks; SevereAt: Severity of attitude to the risks; Envtalsat: Environmental satisfaction; SocialReg: Social recognition; Jobsat: job satisfaction; OHStrained: Occupaitonal and safety tranining.

Figure 3 represents the final model of SEM conducted to determine the correlation of independent variables that were significant at multivariable stages (Tables 2, 4) for knowledge about and attitude toward OHRs.

Table 5 summarizes the significant values of the SEM output. SEM showed that OHS training (*β*: 0.35; 95%CI: 0.27, 0.44), IPC compliance (*β*: 0.07; 95%CI: 0.02, 0.12), environmental satisfaction (*β*: 0.18; 95%CI: 0.01, 0.36), and job satisfaction (*β*: 0.19; 95%CI: 0.03, 0.35) were positively associated with knowledge about OHR perceptions. However attitude level (*β*: −0.121; 95%CI: −0.172, −0.071) and job stress (*β*: −0.248; 95%CI: −0.335, −0.161) were negatively associated with knowledge about OHRs.

**Table 5:** Summary of variables (exogenous) that were statistically significant at SEM analysis for knowledge and attitude (endogenous) toward occupational health risks among SWs.

Endogenous (knowledge and attitude) and exogenous (independent variables)	Observed information matrix (OIM)				95% confidence interval	
	Coef. (β)	Std error	z	P > z	Lower limits	Upper limits
Knowledge (endogenous)						
OHS training	0.35	0.04	8.42	0.000*	0.2	0.44
IPC compliance	0.07	0.02	2.94	0.003*	0.02	0.12
Environment satisfaction	0.18	0.10	1.84	0.050*	0.01	0.36
Job satisfaction	0.19	0.08	2.31	0.021*	0.03	0.35
Job stress	−0.25	0.04	−5.60	0.000*	−0.34	−0.16
Attitude level (endogenous)						
PPE compliance	0.14	0.05	2.78	0.005*	0.04	0.23
OHS training	0.19	0.06	−3.29	0.001*	0.09	0.58
Severity of risk	0.62	0.03	20.71	0.000*	0.57	0.68
Susceptibility of risk	0.29	0.03	9.91	0.000*	0.23	0.35
Social recognition	−0.21	0.05	−3.87	0.000*	−0.32	−0.10

*Statistically significant at 0.05 at the SEM analysis. Goodness-of-fit statistics value of R^2^ for knowledge and attitude was 27.11 and 13.84%, respectively, and overall R^2^ for the model was 33.57%.

In addition, PPE compliance (*β*: 0.14; 95%CI: 0.04, 0.23), OHS training (*β*: 0.18; 95%CI: 0.07, 0.57), perceived severity of risk (*β*: 0.62; 95%CI: 0.57, 0.68), and perceived susceptibility of risk (*β*: 0.29; 95%CI: 0.23, 0.35) were positively associated with attitude toward OHR perceptions. However, social recognition (*β*: −0.21; 95%CI: −0.32, −0.10) was negatively associated with attitude toward OHR perceptions.

## Discussion

The present study found that approximately 67% of SWs working in public hospitals of eastern Ethiopia had insufficient knowledge about OHR. This indicates that almost three-fourths of SWs had poor knowledge of OHR perceptions in these hospitals. This value was slightly less than that calculated across Ethiopia (77%) (27). This discrepancy might be due to the cutoff of the assessment tool. In the present study, the cutoff was three levels, but in a previous study, the cutoff was two levels. However, the finding of the present study is slightly similar to that obtained from a tertiary hospital in Nigeria (65.2%), where respondents had some awareness about OHRs (30). This suggests that the majority of SWs were not well informed about OHR perceptions and could be easily harmed by hazards. Since prior research indicates that people underestimate potential risks or hazards in the workplace due to cognitive bias, a low or high prevalence of low risk awareness could have an impact on how susceptible individuals suffer from those risks (2, 58–60).

Multilevel ordinal logistic regression analysis was carried out to determine the level of knowledge about OHRs and independent variables. It found that SWs who obtained OHS training were more likely to have nearly five times higher level of knowledge about OHRs than those who did not. This indicates that OHS training contributes to enhancing the knowledge of workplace risks among SWs. On the other hand, those better informed about OHRs were more likely to have higher OHR awareness than those who did not obtain training within the hospital. In addition, SWs who were satisfied with their job were more likely to have nearly two-fold higher level of knowledge about OHRs as compared to those who were not satisfied with their job. Furthermore, those who were satisfied with their work environment were more likely to have three times higher level of knowledge about OHRs. A previous study also supports a hypothetical regression analysis, in which workers who were happy with their job and working environment were more likely to have a higher knowledge level of occupational risk prevention within their workplace (61). This is because in a comfortable work environment, people can easily recognize the risk, as reported by Abiodun et al. (30).

This study also aimed to assess SWs’ attitude toward OHRs in public hospitals of eastern Ethiopia. More than two-fifths of SWs had an unfavorable attitude toward OHRs, which indicates that they have a low level of severity of risk perceptions and a tendency to engage in significant amounts of risky behavior. In contrast, this value is 13.5% higher than that obtained from a tertiary hospital in Nigeria, where respondents reported low OHRs (30). This disparity might be attributable to OHS training offered to SWs in Nigerian hospitals, which was not provided in hospitals included in this study. In addition, the perception of susceptibility and safety among SWs toward OHRs was approximately 50%, implying that nearly half of them were unaware of the susceptibility and safety of risk perceptions in their work environment. Multivariable multilevel ordinal logistic regression model showed that SWs with positively perceived susceptibility of risks were more likely to have 10.44 times higher OHRs than those with negatively perceived susceptibility of risks. This indicates that higher levels of perceived susceptibility of risks are related to a lower tendency to engage in risky behavior. The model also showed that SWs with positively perceived severity of risks were more likely to have 48.14 times higher perspectives of risks than those with negatively perceived severity of risks (Table 4). Similar to the above explanations, those with a high level of perceived severity of risks have a lower tendency to engage in a risky behavior. Furthermore, those satisfied with their work environment were more likely to have 2.67 times higher OHRs than those dissatisfied with their work environment. This suggests that there is a correlation between an employee’s happiness with their working environment and OHR perceptions. This hypothesis is consistent with another study, where workers who were not happy with their working environment were more likely to have occupational risks due to the low tendency of risky behavior (61). Finally, SWs who had good IPC practice showed 20.43 times higher OHR perceptions than those who followed poor IPC practice. This indicates that risk perception among SWs was associated with the lack of prevention measure (62).

Regarding to goodness-of-fit-model of knowledge along with predictors was demonstrated. Accordingly, The LR of the multilevel ordinal regression model found that the deviance of model 3 from model 0 was 0.192. In addition, sensitivity of multilevel ordinal logistic regression was 60.22%. This indicates that the output and conclusions were robust and reliable. The square of correlation between the model’s predicted values and the actual values of outcomes of this correlation was 23.43% (pseudo-*R*^2^ = 0.2343), Besides, the Hosmer–Lemeshow goodness-of-fit, the *p*-value was 0.30 (HL goodness *X*^2^ = *p*-value = 0.30), it was greater than 0.05, then fails to the null hypothesis. In same analysis the goodness-of-fit-model can explained using the values of model out put found under (Table 4).

Furthermore, SEM showed the correlation of knowledge and attitude with their explanatory or independent variables. In this model, positive (+β) and negative (−β) values were generated during analysis. Accordingly, +β indicates that the increment of independent variables enhances the level of knowledge about OHRs. For example, compliance with IPC practice was significantly positively correlated with SWs’ level of knowledge about and attitude toward OHRs. This finding is consistent with the findings of a previous study (63), which claimed that the presence of PPE probably has a positive effect and can reduce exposure to risks. Another possible explanation is that adhering to safety measures such as IPC practice and available PPE could increase the level of knowledge, which leads to a lower tendency of risks (64).

SEM also revealed that OHS training was significantly positively correlated with SWs’ knowledge about and attitude level toward OHR. This finding is slightly similar to the finding of a previous study (65), where SWs who were trained on OHS service had high knowledge about OHR perceptions. In addition, job satisfaction was significantly positively correlated with the level of knowledge about OHRs. The possible explanation is that good job satisfaction has a direct relationship with good knowledge about OHRs among SWs within the hospital. Another study also reported this scenario, where job satisfaction reflected on overall quality of life, showing that knowledge of perceived health status prevents serious psychological conditions in the workplace (66).

However, −*β* indicates that the impact of the variable has the potential to decrease the level of knowledge about and attitude toward OHRs. In this study, the level of unfavorable attitude toward OHR decreases with the level of knowledge about OHRs. The findings of this study are consistent with those of a previous study (67), where negative (lower) attitude was significantly associated with low knowledge among individuals at the workplace. Furthermore, social recognition was negatively associated with the attitude of SWs toward OHRs. This suggests that poor social recognition can lead to a low degree of attitude regarding OHRs. Because the study found that good social recognition at workplace, clearly demonstrating how significant visibility for well-being of workers at work in order to lower the risks (68).

## Strengths and limitation

### Strengths of the study

This study used a cross-sectional design, which allowed for the simultaneous collection of all data. This led to completing the interpretation of the findings and associated factors in a short period of time. In addition, this study provided strong evidence in favor of descriptive analysis and formulation of research hypotheses on SWs’ knowledge about and attitude toward OHRs.

### Limitations of the study

Despite its advantages, this study did not measure incidence, associations identified may be difficult to interpret, and it is susceptible to bias due to low response, misclassification due to recall bias, and non-response. In addition, this study followed a cross-sectional design, which might have resulted in less information to make a causal inference and inability to investigate the temporal relationship between knowledge and attitude toward OHR. Moreover, there were only a few pieces of evidence found worldwide regarding knowledge and attitude toward OHRs, particularly in hospital and healthcare settings.

## Conclusion

This study concluded that SWs’ lack of knowledge and experience regarding OHRs could result in claims related to health and safety in hospital settings. It also concluded that the majority of SWs had a negative attitude toward OHRs, which might have resulted in job-related impairment. As a result, the following intervention strategies can be implemented: increasing SWs’ awareness, promoting safety precautions such as PPE, offering OHS training, fostering a positive work culture to reduce SWs’ negative perceptions, establishing an IPC environment, and carrying out routine monitoring. The study also suggests that hospitals offer advisory services regarding knowledge and attitude in line to improve occupational health risk prevention to lower workplace risks among SWs sufferers. Furthermore, this study also advises that policymakers keep policies in place to improve knowledge about and attitude toward OHRs by encouraging safe practices, offering safety PPE, conducting frequent training, providing sufficient reinforcement, and enhancing the potential of SWs, which can reduce the tendency of OHRs.

## Data Availability

The raw data supporting the conclusions of this article will be made available by the authors, without undue reservation.
