# Supplementary material for: Occupational health risk perceptions and determinants: knowledge and attitude of sanitary workers in public hospitals of Ethiopia
Source: Front Public Health. 2025 May 9;13:1509595. doi: 10.3389/fpubh.2025.1509595 (PMC12098515; doi:10.3389/fpubh.2025.1509595)
Supplement: Supplementary file 2 [file Data_Sheet_2.PDF]

ANNEX: ASSESSMENT TOOLS

English language version Competent Adults: Ages > 18 Years)

1. Introduction:

My name is ___________________________________, I am working as a data collector for the study being conducted in this community by Sina Temesgen Tolera, Tesfaye Gobena, Nega Assefa, Abrahama Gremew and Elka Toseva. I kindly request you to lend me your attention to explain you about the study and being selected as the study participant.

2. The study/project title:

Occupational Health Risk Perceptions and determinants among sanitary workers public hospitals in eastern Ethiopia

3. Purpose/aim of the study:

The findings of this study can be of a paramount importance for the hospitals and other sectors to plan health and safety practice in the save environment among sanitary workers namely cleaners, waste collectors and others. Moreover, the aim of this study is to write a dissertation as a partial requirement for the fulfillment of a Doctor of Philosophy's Program in Environmental Health for the principal investigator.

4. Procedure and duration:

I will be interviewing hospital sanitary workers, namely cleaners, waste collectors and sewage workers using a questionnaire and physical observations to provide me with pertinent data that is helpful for the study. There are 80 questions to answer where I will fill the questionnaire by interviewing them. The interview on each hospital sanitary worker will take about 45-60 minutes.

5. Risks and benefits:

The risk of participating in this study is very minimal, but only taking few minutes from sanitary workers' time. There would not be any direct payment for participating in this study. But the findings from this research may reveal important information for the hospital particularly amendment of occupational health and safety guideline, and risk mitigation, which is one part of infection prevention activities in the hospital.

6. Confidentiality:

The information that we will be provided will be kept confidential. There will be no information that will identify the participants in particular. The findings of the study will be general for the study community and will not reflect anything particular of individual persons. The questionnaire will be coded to exclude showing names. No reference will be made in oral or written reports that could link participants.

7. Rights:

Participation for this study is fully voluntary. The participants have the right to declare to participate or not in this study. If they decide to participate, they have the right to withdraw from the study at any time and this will not label them for any loss of benefits which they otherwise are entitled. They do not have to answer any question that they do not want to answer.

8. Contact address:

If there are any questions or enquires any time about the study or the procedures, please contact: Sina Temesgen: sinatem3@gmail.com; +251913023634; Institutional Health Research Ethics Review Committee (IHRERC) at office phone 0254662011 or P.O.Box 235, Harar, Ethiopia

9. Declaration of informed voluntary consent:

I have read/ was read to me the participant information sheet. I have clearly understood the purpose of the research, the procedures, the risks and benefits, issues of confidentiality, the rights of participating and the contact address for any queries. I have been given the opportunity to ask questions for things that may have been unclear. I was informed that I have the right to withdraw from the study at any time or not to answer any question that I do not want. Therefore, I declare my voluntary consent to participate in this study with my initials (signature).

1. Name and signature of participant: __________________________Date ___________
2. Name and signature of Data Collector: _________ ___________ Date __________

N.B

This is signed face to face in the presence of the data collector.

Please provide a copy of this signed consent to the participant.

If the participant is a lay person and cannot sign initials, can put his/her thumb print in front of a competent witness; and the witness has to sign alongside (with his/her name and address).

S.n	1. Socio demographic characteristics (Participants Code:__________)						
	Code: ________						
01	Employment: 1 Permanent ( ) 2. Contract ( ) 3. Outsourced ( ) 4.Other ( )						
02	Sex	Male ( )	Female ( )				
03	Age: ______________________						
04	Work Experience ______________________						
05	Educational status ______________________	___________					
06	Marital Status (Tick√) Single ( ) Married ( ) Separated ( ) Divorced ( )						
07	Income monthly salary: ________________						
08	Job categories (Tick√): Cleaners ( ) Waste Collectors ( ) Waste emptier ( )						
	Type of your shift [Job rotation]? _____ 1st shift( )____2nd shift_( )_3rd shift_ ( ) There is no Shift ( ) _____						
5.Perspective of the workers							
A)	Perspective 1: Knowledge (Tick under your response)	Yes	No				
9	Do you know the chance of hepatitis infection due to contaminated waste?	Yes	No				
10	Do you think needle stick injury is one of your occupational risks?	Yes	No				
11	Do you know that the hospital facility is highly infectious	Yes	No				
12	Do you know pieces of cloth and metal, used syringes and needles, soiled gauze and cotton are the causes for occupational disease?	Yes	No				
13	Do you know about occupational health and safety service at your settings	Yes	No				
14	Do you know the precautions of safe disposal for needles and any sharp wastes?	Yes	No				
15	Do you know occupational health hazards could due to unsafe working conditions	Yes	No				
16	Do you know nosocomial infections can be transmitted through blood and body fluid contamination while you working?	Yes	No				
17	Do you think pulling and pushing the heavy materials could be cause MSDs?	Yes	No				
18	Do you think work load and beyond normal capacity could be resulted occupational problems?	Yes	No				
B)	Perspective 2: Attitude: Tick [√] under your response : (Where: 1:Strongly, 2:Disagree, 3:Neutral Disagree; 4: Agree; 5:Strongly Agree*)*		1	2	3	4	5
19	I believe my chances of developing an occupational illness are great						
20	I feel that I have good chance of getting an occupational illness in my career						
21	I know people in this career field who have an occupational illness						
22	I am aware of post exposure prophylaxis for the prevention of biological hazard						
23	I believe that following standard precautions like PPE can decreases the work risk						
24	Training can reduce risk from occupational health and safety						
25	The thought of getting an occupational illness is deeply concerning						
26	If I developed an occupational illness, my career would be in jeopardy						
27	I believe I'm free from occupational health and safety hazards.						
28	I believe the chances of OHS hazards are relatively small or even insignificant						
6.Other related factors of OHS outcomes							
29	Did you get occupational and health safety training yet?	Yes	No				

30	Do have sleep disorder/disturbance due to work related problems?	Yes	No
31	Currently, do you consume an alcohol? [If Q42 “No” go to Q43].<br>If Yes, what is frequency?<br>Seldom /Monthly ( ) Occasionally/Weekly ( ) Frequently/Daily ( )	Yes	No
32	Have you currently have work load? [If Q43 “No” go to Q44]<br>If “YES”, hours/week?<br><40hr/week ( ) 2. 40-45/week ( ) 3. Greater than 45hr/week ( )	Yes	No
33	Do you currently work more 8 hr/day? [If Q44 “No” go to Q45]<br>If “YES” what is frequency?<br>1) Seldom /Monthly( ) 2) Occasionally/Weekly( ) 3) Frequently/Daily ( )	Yes	No
34	Do you currently chew khat? [If Q45 “No” go to Q46]<br>If “YES” what is frequency?<br>1. Seldom /Monthly ( ) 2. Occasionally/Weekly ( ) 3. Frequently/Daily ( )	Yes	No
35	Currently do you smoke tobacco? [If Q46 “No” go to Q47]<br>If “YES” what is frequency?<br>1) Seldom /Monthly( ) 2) Occasionally/Weekly( ) 3) Frequently/Daily( )	Yes	No
36	Do you have job stress? [If Q46 “No” go to Q47]<br>If “Yes” how you feel?	Yes	No
	1: Never ( ) 2: Low ( ) 3: Middle ( ) 4: Serious ( ) 5: Very serious ( )		
37	How you satisfy with your current job? [If Q48 “No” go to Q49]<br>If “YES” how evaluated it?<br>1: Very dissatisfied ( ) 4: Satisfied ( )<br>2: Dissatisfied ( ) 5: Very satisfied ( )<br>3: Neutral ( )	Yes	No
38	How you satisfy with the work environment? [If Q49 “No” go to Q50]<br>If “YES” how evaluated it?<br>1: Very dissatisfied ( ) 4: Satisfied ( )<br>2: Dissatisfied ( ) 5: Very satisfied ( )<br>3: Neutral ( )	Yes	No
39	Is there social recognition for your work? [If Q50 “No” go to Q51] If “YES” how other professions including managers perceived you?<br>1 Good ( ) 2. Bad( )	Yes	No
40	Are you obeying personal protective equipment practice during your work?	Yes	No
41	Do you practice infection prevention and control activities (safe disposal of medical waste) within the hospitals	Yes	No

Thank you very much for you in advance!
